# Meeting Sojourner at the Intersection: Women of Color Living and Aging with HIV

**DOI:** 10.3390/healthcare13111280

**Published:** 2025-05-28

**Authors:** Denise Torres, Jade Marie Nesbitt, Sharlene Allen-Milton, Laurens G. Van Sluytman

**Affiliations:** 1 The Murphy Institute, CUNY School of Professional Studies (SPS), City University of New York (CUNY 1), New York, NY 10001, USA; 2 School of Social Work, Morgan State University, Baltimore, MD 21251, USA

**Keywords:** aging, Sojourner syndrome, intersectionality, older adults, healthcare utilization, resilience, HIV/AIDS, gender, structural violence

## Abstract

**Background/Objectives:** Women of color remain at risk of new HIV diagnoses. This study applied an intersectional framework to explore the lived experiences of women of color aged 50 and older who are living and aging with HIV. **Methods:** The researcher conducted a secondary analysis of data from a study involving respondents aged 50 years or older living with HIV. The mean age of the female participants (N = 12) was 57.42 (SD = 5.18, range = 47–65). Ecological Systems Theory was used to operationalize intersectionality, considering participants’ multiple identities and social locations. **Results:** Participants described histories of role enactment and struggles as they faced structural, community, and interpersonal violence, anticipatory loss, and shame. Their narratives highlighted how layered oppressions shaped their experiences across the life course. **Conclusions:** Findings underscore the importance of using intersectional theoretical frameworks to examine the intersecting systems of oppression affecting older women of color living with HIV. The study recommends trauma-informed assessments and interventions, as well as culturally informed clinician training rooted in evidence-based practices.

## 1. Introduction

Despite reductions in new HIV infections, women of color—especially those who identify as black/African American—remain at considerable risk, representing almost 60% of new diagnoses [1]. Critics have noted that women of color enact and navigate their multiple locations and myriad oppressions while living with HIV/AIDS. This is often ignored, continuing the essentialization of their identities and experiences [2,3]. This also includes how their categorical risk and vulnerability are conceptualized, how their experience is researched and analyzed [4], and how intersectionality theory is employed [5,6]. These points are especially salient as optimal treatments and illness management impact health and wellness outcomes. By illuminating how older women of color living with HIV/AIDS remain resilient, this project attempts to respond to these gaps.

The secondary analysis data of Generation Boom: Sustainable Protective Behavioral Norms Related to HIV Medical Care and ART Medication Therapy Adherence among HIV + Americans Aged 50 Years and Older [IRB #17/09-0119] captures the project’s unanticipated themes. Theoretically, Ecological Systems Theory (EST) is used as the skeleton, the bones upon which are laid the Sojourner Syndrome [7,8], the connective tissue and flesh embodying respondents’ situatedness. The literature review highlights how EST is integral to conducting intersectional examinations of how individuals maintain safety and perform various aspects of their identity. Mindful that the salience of any particular category is situational, the analysis draws from extant constructs and concepts. While these are numerous, they facilitated the most sensitive and descriptive rendering of the complexity of the women’s lived experiences and how their identities are enacted, allowing us to “do” intersectionality in ways that privilege their voices.

### 1.1. Literature Review

Because most theories inadequately address issues of race/ethnicity, cultural competence, or social inequities, social workers have been called to incorporate critical race theory (CRT) [9,10,11]. As implied, “crit” theories critique traditional theoretical approaches for neglecting issues of power and historicity as well as their acceptance of the status quo and, therefore, center knowledge production for emancipation and liberation and systems change. Emerging from US legal scholars, CRTs expose how racism (and other discrimination) is integral to socio-legal structures, challenge the concept of “color-blindness”, and promote reflexivity [12,13]. Responding to the constructed, historical, socio-political, contextual, and interactional mechanisms of oppression embedded in systems, intersectionality helps to avoid serving as a passive enabling servant [14] to those same forces. It is thus deemed essential to research and practice aiming to address disparities in health outcomes for marginalized communities [15].

### 1.2. Intersectional Theory and Praxis

As a meta-theory, intersectionality permits and draws from multiple other theories and frameworks. In particular, it demands knowledge of General [16] and Ecological Systems Theories [17], which are central to social work and public health practice [18,19]. They help define service (i.e., help people and address social problems [20]). Despite being assailed as too “mechanistic” and unresponsive to power dynamics, systems proponents underscore the difficulty of observing and quantifying the “transcendental” and “symbolic” and decry a demand for “neat formalism” by “dogmatists” [16,17,18]. Rather than static renderings of inert parts, the system theory emphasizes the transactional nexus between subsystems that maintain the whole. However, the more complex a system (i.e., human socio-organizational and transcendental), the greater the difficulty of deriving explanatory, universal theories about it. These interactional fields are rife with diverse and intangible exchanges, such as relationships (both as interrelations and roles), symbolization, time, and “the content and meaning of messages ... and value systems” [20] (p. 205). Indeed, to capture the adaptability of human systems [17] incorporated the chronosystem—accounting for time and historical contexts’ impact. Thus, intersectional and systems theories center on historicity, context, and interaction.

Intersectional praxis demands emancipatory interventions directed at and across multiple systems, given that the processes and attendant meanings and values that marginalize and oppress have changed very little. Intersectionality recognizes the reciprocal nature of oppressive systems: they morph, co-opt, and adapt to counter attempts to equalize the playing field or, in systems language, they maintain an “exchange balance” [18]. Intersectionality views this maintenance of injustice as pernicious. Whether violence is behavioral, as discussed in Crenshaw’s [12] seminal analysis which dealt with domestic violence, or structural, such as the inequality of basic necessities such as shelter, food, and healthcare [21], it remains embedded in systems exploitation and resources competition. Furthermore, structural violence limits the individual’s potential to participate fully and/or survive as long as each member could be expected to live if total resources were reallocated [22]. As such, political action is imperative as these are the processes by which individual and collective progress is undermined. As praxis, intersectionality aligns completely with social work’s responsibility to the broader society [20].

Intersectionality is process-oriented and uncommitted to particular subjects or identities even as it focuses on structures of power [13]. This is important to note given that Crenshaw’s [23] articulation has traveled and evolved such that intersectionality has become a “buzzword” [24]: a simple mention of race/ethnicity, class, and gender or a quick and dirty cross-tabulation is deemed sufficient to have “done” intersectionality. In the US, these core categories continue to mark differential vulnerability, but they are inseparable and contextual rather than discrete aspects of identity. The salience and potential privilege of any category are dynamic, shifting, and dependent, with intersectionality as a means for “marking and mapping the production and contingency of both” [13] (p. 815). That is, the power of intersectionality is not in its ability to identify ever more categories or issues to map on a matrix, but to focus analytic and clinical lenses on their layered expression. Hence, “doing” intersectionality requires an examination of how identities are performed and enacted across various systems at various times.

Much of the intersectional literature is dedicated to examining how identities are experienced and expressed contextually. This broad literature uses terms such as “working”, “negotiating”, “enacting”, “performing”, and “managing” identity [13,25,26,27]. Identity performance or performativity assumes that individuals are active agents who assess and then enact identity to best fit the circumstances. Performativity draws from Goffman’s Dramaturgical Perspective [28], which posits that individuals act to achieve personal and cooperative ends through socially acceptable means; these “dramas” allow actors to fulfill needs in standardized ways, with actors using “fronts” and “masks” to communicate the socially constructed and symbolic nature of interactions within a cultural space or “performance”. Such cooperative performances assist individuals to avoid shame and maintain boundaries. A means of mediating interactions, performances create a standard currency—a lingua franca—that allows sufficient intimacy to maintain social cohesion, although they perpetuate status differentials between and amongst individuals and groups as the exchanges become routinized and stylized. Hence, while performances allow actors to maintain their individuality and self-integrity, their membership in a class or group is reinforced, tropes are perpetuated, and stereotypes are reified.

Critical to understanding the lived experience of this study’s respondents—women of color living with HIV/AIDS—is the intersectional interpretative framework entitled the Sojourner Syndrome, which posits that African American (AA) women cope actively with stressors resulting in higher rates of morbidity and mortality [29,30]. Emerging from HIV/AIDS research with African American/black women, the Sojourner Syndrome highlights the reciprocal and interactive effects of race, gender, and class in structuring their HIV risk as well as protective factors such as kinship. Named after the former enslaved woman and champion of abolition and women’s rights, Sojourner Truth, the framework captures how African American/black women respond to class exploitation, gender subordination, and racial discrimination by adhering to gendered roles (e.g., family and community responsibilities) while also taking on primary responsibility for work outside as well as heading households [29]. This survival strategy has assured the continuity of black communities across four centuries of oppression and violence.

The consequences of enacting the Sojourner Syndrome across a lifetime in terms of work and shadow work wears down or weathers women. First posited by Geronimus [7,31], weathering views socioeconomic disadvantage as a cumulative stress embodied in health statuses and outcomes among black women, particularly concerning maternal and child health. This biopsychosocial model links and theorizes the role of stress physiology, molecular dynamics, allostatic loads, and mechanistic pathways [32], underscoring the silently corrosive effects of exposure to stress. That the stress of marginalization and oppression is deleterious to a population’s health is demonstrated by the fact that even when controlling for socioeconomic and access-related factors, racial/ethnic minorities in the US receive lower quality care and are at greater risk for potential diseases. First conceptualized among women of childbearing age, it has since been extended to older women [33], men [34], and other groups, even as differences in weathering patterns among African Americans reaffirm that black communities experience discrimination differently even as compared to other racial and ethnic groups [35]. If, as structural violence theorists posit, health and longevity reflect a society’s stratification and indicate the level of structural violence within it, then African American women are disproportionately situated as recipients of the emanating cumulative waves of the society’s assaultive forces.

The Sojourner Syndrome provides a historical narrative and ritualized performance through which the researchers can sensitively interpret respondents’ meaning-making of interactions and experiences across the various systems within which respondents report negotiating their identities. This interpretive framework effectively centers its attention on those transactional performance spaces, wherein processes of oppression and responses to them may begin to be mapped, specifically, at the meso- and microsystems. Furthermore, the Sojourner Syndrome proposes that the intensive work needed to perform in these spaces impacts long-term well-being through acculturative stress or weathering. The importance of the chronosystem is not only to capture the health consequences over time for the individual but also to mark significant cultural moments (e.g., 9/11 and the death of Martin Luther King Jr.), which continue to resonate across a lifetime. Lastly, as will be explored in the discussion and implications sections, this schematic identifies and focuses our attention on those systemic forces on which women of color have little influence but significantly impact how they work their identity.

## 2. Materials and Methods

This study uses data from a more extensive study conducted between January and March 2019, which engaged 25 Americans, 50 years of age or older, living with HIV in using a technology-assisted CASE Adherence Index [36]. The CASE Adherence Index, predictive of virologic responses and highly correlated with previously validated instruments [37] is a composite tool comprising three self-reported measures of adherence: the frequency of difficulty taking HIV medications on time, average number of days per week at least one dose of HIV medication was missed, and last time missed at least one dose of HIV medications. The CASE Adherence Index can be employed to measure comorbid and non-HIV prescriptions at monthly intervals. As part of the second prong of the research, at 3-month intervals, the relationships between sociocultural-level factors; predisposing, enabling, and need factors; protective behavioral norms (e.g., substance use, diet, service utilization) related to HIV medical care; and ART medication therapy adherence were explored using a phenomenological approach. A discovery-oriented method, phenomenology enhances the exploration of the inherent logic [38] constructed by participants regarding sociocultural-level factors; predisposing, enabling, and need factors; protective behavioral norms related to HIV medical care; and ART medication therapy adherence [36,39]. Because phenomenology declares that no one perception is right or more accurate than the other [40], facts are established within a value framework, phenomena are contextualized, and data are approached without premature theories, provisional hypotheses, or tentative speculations. This secondary data analysis emerged abductively and evolved during the coding and memoing process.

### 2.1. Sampling and Data Collection

The project employed purposive sampling to ensure all participants had direct experience with the phenomena under study. Thus, all participants were aged 50 years or older and had been diagnosed with HIV. Participants were recruited from medical and community health organizations. Ethical safeguards, including informed consent, confidentiality measures, and distress management protocols, are in place to protect participants while minimizing risks, such as emotional discomfort or fatigue. This study was approved by the Morgan State University Institutional Review Board (IRB). The primary sample size (*N* = 25) adhered to the recommendation of numerous qualitative researchers—a small sample allows for repeated contact and greater involvement [41,42,43] to illuminate the relationship between salient characteristics and pertinent social circumstances. This sub-sample represents all of the women engaged in the research project. The semi-structured qualitative interview, conducted by the project’s principal investigator, used one demographic questionnaire and a semi-structured interview guide to conduct individual interviews with participants who consented to be interviewed. Each interview lasted between 45 min and one hour and resulted in a transcript of between 25 and 50 pages. Participants were compensated USD 25 at baseline and at each subsequent interview; participants were followed for 6 months to determine their adherence to medication regiments, all of which was digitally recorded for transcription and analysis.

### 2.2. Participants

The mean age of female participants (*N* = 12) was 56.39 (*SD* = 5.234, range = 47–65). (Table A1). Though the primary project focused on HIV + Americans aged 50 years and older, Table A1 provides the sample’s characteristics for only the female participants. Eighty-three percent of participants identified as non-Hispanic–African American or black. Furthermore, 8% were between 40 and 49 years old, 50% were 50–59 years old, and 42% were between 60 and 69 years old. Most (92%) live in their own homes. While 8% indicated having completed the eighth grade or less, over 50% reported completing a high school equivalency course or some college or technical school. The majority, 58%, received Supplemental Security Income or SSI payments or Social Security Disability Insurance (SSDI) as a source of income. However, 50% indicated that they were employed during the last six months. Though 83% reported having some source of health insurance, 17% reported having no health insurance. One hundred percent of respondents scored within normal limits on the Rosenburg Health Status Scale at the time of the interview.

### 2.3. Data Analysis

For this secondary analysis, the authors engaged with the transcripts of the 12 female respondents. First, the research team familiarized themselves with the data and their content through reading and re-reading the transcripts. Next, the PI and co-author conducted independent coding to identify patterns in the data, yielding seven codes—such as economic instability, access to basic needs, navigating disclosure, and stigma; the next step, axial coding, sought to identify relationships between codes. Thus, economic instability and organizational/bureaucratic barriers were combined to form structural violence. Specific quotes were then selected to illustrate the social processes discussed by the participant as identified by age and race/ethnicity. Such descriptors add depth to the conceptual areas concerning similarities and differences. The project team met to refine and validate these. Each of these was defined based on participant narratives. For example, Shame and Stigma emerged from responses like, “I didn’t tell nobody until 2008 because I thought my family would look at me differently” (61 years old, diagnosed UNK).

## 3. Results

The data analysis of the transcripts revealed active responses to counter and manage identities as women across multiple life domains. The narratives they presented were complex, often weaving in and out of the past and into the present as respondents allowed the researchers into their unique world and the strategies employed to maintain safety and wellness for themselves and their families. Indeed, during multiple instances, the interviewer probed and clarified to ascertain if an event occurred during the recent or remote past. While the domain of focus may have differed by respondents, a consistent subtext throughout all interviews with female respondents was the attempt to maintain power, agency, and self-integrity when they experienced a threat to their sense of self. Consistent with the integrated interpretative framework offered above, the researchers present how the women enacted and struggled to perform their roles at the interstices of the micro- and mezzo-community and macro-systems, along with the structural, community, and interpersonal violence they navigate across time. Furthermore, the researchers offer that the strategies they appear to implement at particular levels defend against anticipatory loss and shame.

### 3.1. Structural Violence

Economic stability and safety were consistent themes among respondents, regardless of their source(s) of income or history of employment. If, as Kohler and Alcock (1976) [23] suggest, inability of meeting basic needs represents a form of structural violence, then each respondent offered a concrete story of the difficulty of survival. Across time and life domains, each spoke to either a history of struggle or current concerns regarding meeting basic needs, including food, shelter, and employment. For example, one respondent offered that in her childhood

*food was, I guess, I don’t wanna say it was scarce, I guess it depends on family and how the parents provided. Because, you know, back then, it was, you know it was also food stamps back then* (55 years old, diagnosed 2004).

This narrative offers not only information pertinent to the individual contextual level but also at the familial level and its engagement with larger systems and time. Similarly, and somewhat nostalgically, a respondent suggested there was less community struggle because of the safety net, although it was her greatest challenge today. She stated there was “*Plenty of food, because people that was on, at that time it was welfare. They got that supplement food* [but today] *makin’ sure that my grandkids have a place to live, food to eat, clothes on their back*” (64 years old, diagnosed 1991). Others affirm the value of engaging with charitable organizations as they struggle to attain basic needs. Another participant (54 years old, diagnosed 2001) noted the following:

*Not clothin’, but food, not food and clothin’. I’m supposed to get furniture in March from Silver Springs, Maryland. My friend, uh, social worker hooked me up with [agency providing free furniture]*

However, participants also reflected on the impact of contractions in welfare entitlements and their impact on one’s capacity to obtain sometimes the most basic of life’s necessities, such as housing and medical services:

*Oh, housing, it took me years to get housing. I only got housing because I took my niece in for a little bit. And because I took my niece in the room she, I needed more room, the social worker helped me housing, Section 8 housing.* (52 years old, diagnosed 1987).

Furthermore, the lack of consistent insurance coverage and/or bureaucratic red tape often interferes with making more informed decisions regarding treatment courses, with one respondent stating clearly such discussions were on *“the back burner right now, ’cuz first I need to get insurance”* (56 years old, diagnosed UNK).

Yet, respondents still spend significant time and energy attempting to get systems to serve them fairly and respond. One respondent explained how, even after four years and multiple appeals, she was still being harangued for money she did not owe:

*… so that makes you ineligible for the check. But then they told me when they cut the check that I could still have Medicare Part A and B, as long as I paid the premium, ... It took me 4 years to win that case with um, lawyer from [local legal services center], of course they did pro bono work. I took it all the way to their judge, there* (52 years old, diagnosed 1987).

Within the world of work, respondents’ identities resulted in complex discriminatory experiences. One respondent, a campus police officer, spoke to persistent gender role constructions and racial tokenism, describing how her sense of agency and personal power was undermined as a supervisor

*felt as though I belonged home, barefoot and pregnant, and takin’ care of some man. And I said, can I speak frank sir, I said, ‘what the f-ck you hire me for then’. And he said, ‘that’s how uh, the census bureau determined that we needed an African American, black woman and fit the bill’. And that’s all I was there for* (55 years old, diagnosed 2005).

Attempts to confront and address this via the correct organizational channels resulted in further harassment, alienation, and, ultimately, emotional exhaustion, such that she withdrew. The respondent explained

*I tried to complain and, my captain came at me more bad, because he said I was wrong for filin’ a complaint against him, ’cuz he sexually harassed me too. And I was wrong. I went against the grain. The ‘bro’ code of a officer, filin’ a complaint against another office, and that’s what finally made me resign, ’cuz I couldn’t take it no more* (55 years old, diagnosed 2005).

Respondents’ narratives of their exposure to violence must be understood within a context that examines the unique experience of the environment and participants as well as the interactive processes informing outcomes. The denying of agency to a female officer who had risen through the ranks, forcing her to resign, has a significant impact on the presence of African American officers in African American communities, often disproportionately beset by poverty and an increased exposure to violence, as discussed by the following respondent.

*I live in an apartment beautifully well-kept lawns, but there’s still shootings. There’s still a lot of shootings and a lot of things are goin’, when I use to worry about gettin’ sick, I’m also worried about gettin’ shot. Being sick or being in some really bad situation because of the violence in Baltimore. So I really worry a lot about my kids, and you know, and myself as far as the violence part. And this world is just* (52 years old, diagnosed 2005).

Collectively, these respondents articulate the cumulative as well as the redundant impact of structural violence. However, the following participants capture the historical indignities and the experience of violence women of color have endured as they engage in perhaps the most fundamental of biological processes. The following participant describes the marking of HIV-infected women during childbirth.

*That’s when they did the little red dots, they would do red dots. That would signify to the healthcare staff that you were positive. They wanted to put a big red dot on my door when I had my son with me, and uh, I just, I hurried on, so they didn’t do it. They had to do it another way than to do a dot, ’cuz people were like, what the hell is this red dot doin’ here. And you know um, I had a problem with that* (52 years old, diagnosed 2005).

### 3.2. Shame

Regarding the experiences of heightened vulnerability due to their exposure to violence, participants described the experience of living with the shame associated with the individual experience of living with HIV. Forty years into the epidemic, well into understanding methods of transmission, one respondent described an interaction with her doctor that reinforces both her need to remain sexless as well as her potential to contaminate.

*He turned around and looked at me, and I said, ‘I don’t need no condoms’. I said ‘because I’m not havin’ sex’. And you know what my doctor said to me, he said, good. … So I feel as though when he said ‘good’ that meant for me to not to have sex, and I don’t. … I just don’t even want to talk about it* (62 years old, diagnosed 2001).

#### Anticipatory Loss

Sojourner Truth was said to have possessed the extrasensory skill of second sight. The women in this study appeared to be no less skilled as they encountered interactive systems that repeatedly challenged their right to agency, self-determination, and even humanity. Several respondents indicated that after repeated losses, they are surrounded by confusion. They both speak poignantly about loss on the individual and community level, and on close examination, they speak of a history of loss.

*Well, I’m afraid that most of my peers, I got one good friend left. I’ve been dealin’ with a lot of death lately, because both of my brothers died, my other brother died this year, my oldest brother died this year. He was positive. My youngest brother who was gay, he was positive, he died too. So, I’ve been in a very awkward emotional state with that, ‘cuz I just saw him, and then I just lost my nephew the same year. Um, a lot of thoughts, a lot of confusion, you know what I’m sayin’ around how I feel. And when I feel, I see them slip away, I see myself. You know, I see me* (52 years old, diagnosed 1987).

*This year has been pretty emotional for me. I’m not really feelin’ the holiday thing right now, not because of my HIV, but because of lost people. And I think about how they left too, especially the ones so close to me. Um, so it reflects on me. It reflects on me, so, it’s been an iffy, iffy year emotionally. And most of my peers, my best friend, __, she passed away. I only have one left* (52 years old, diagnosed UNK).

Anticipatory loss and its associated coping methods challenge respondents to remain engaged in their communities. Another participant described retreating from further engagement.

*I’m scared to try to date again, because I don’t know how a person with, like my status, I don’t know. I’m tryin’ to get out there to just to, but I haven’t got to that point yet* (53 years old, diagnosed 1987).

Thus, despite their awareness of methods of transmission, their engagement with medical systems, and their active involvement with HIV treatment and prevention, many women expressed their fear of having to tolerate future loss either through the death of significant others or through rejections. They are resolute in their experience of repeated loss not only of real others, children, friends, and family members but also of identity. This is particularly true for women who have had histories of drug use or sex industry involvement.

### 3.3. Impression Management

To negotiate the alternating challenges associated with multiple roles, e.g., partners, daughters, and parents, among others, respondents discuss methods of managing the impression they convey within existing relationships. Fearing backlash from significant immediate others as well as others in the broader social environment, participants in this study reported engaging in behavior designed to mitigate threats to the self or one’s capacity to access needed resources.

*Because I be scared to tell people I have, because I’ve already seen what it feels like to tell people what I have and how they treat me after they know. So I’m not real good with that right now* (54 years old, diagnosed 2005).

Another participant described the enduring stigma associated with HIV disease. Her description moves from the fear of rejection to making decisions concerning disclosure based on mitigating the threats to identity-based expectations of others. She discusses not feeling herself while denying feeling “dirty”.

*I didn’t tell nobody until, I think I told my mom in 2008. … Because I thought my family would look at me different, and I didn’t want, I didn’t want to tell anybody because at that point, I felt bad. I felt, I wouldn’t say dirty, but I just didn’t feel, I wasn’t myself. I didn’t feel like myself* (61 years old, diagnosed UNK).

The perception of threat and the effort to reduce the impact of expected rejection and prejudice are so real that participants describe a sense of relief when relationships prove to be reliable despite revelations of an HIV status and when others are made aware of the participant’s needs.

*I did not. What I did, because I was pretty much ashamed of the diagnosis. I told them I had a cancer diagnosis. I didn’t tell them I had AIDS or HIV. They know now. A few of them know now, but that’s what I told them at the beginning, that I was very sick, and I had cancer. I did not say AIDS or HIV. Because at that time it was a stigma, I didn’t know how my family would react. Um, but you know, they act like most families, they were supportive when I was sick, but they didn’t actually know the actual diagnosis* (52 years old, diagnosed 1987).

Yet, and still, at the interstices of these interactive processes, participants describe shouldering the expectations of individual, community, organizational, and larger social constructs concerning what is and is not desirable. They rise up to meet these challenges for the sake of obtaining resources to provide for themselves and their families. In so doing, the participants in this study described encounters with shame and anticipatory loss. They also describe a set of identity performances enacted to achieve a sense of integrity.

## 4. Discussion

African Americans and Latinos accounted for 37% and 33%, respectively, of the new cases of HIV infections in the United States of America in 2022 [1]. At the end of 2022, it was estimated that approximately 152,700 Black/African American women were living with HIV, 8 times higher than White women and 3 times higher that Latino women [1]. Though significant inroads have been made in reducing the rates of new infection, the Centers for Disease Control (CDC) reported in 2016 that only 67% of people with HIV, both diagnosed and undiagnosed, received some HIV care, and only 49% were retained in care to achieve viral suppression. Achieving viral suppression contributes to maintaining health as well as reducing the risk of sexually transmitting HIV to HIV-negative partners. Therefore, the access to care and adherence to treatment is a critical element of HIV treatment and prevention. The factors that challenge their capacity to adhere to treatment require more in-depth exploration.

Often, social and public health services are offered as a solution to health challenges. This assumes that treating the illness will alleviate the challenges the individual faces. This assumption has recently been refuted as many women of color, as frontline workers and primary caregivers in their families and communities, faced the ravages of a global pandemic. The individual’s experiences of social hierarchies (e.g., gender, race, and disability) may vary greatly depending upon the woman’s particular circumstances. Our findings suggest that social service public health providers and policymakers attend to the complexity of women of color’s lives and work with these vulnerable populations to prevent further systemic and structural violence while providing opportunities to improve the conditions of their lives. Our analysis demonstrates the considerable strength of theoretical frames that employ intersectionality. Our frame suggests a model of analysis that examines the interactive, multiple social identities and systems of oppression that structure the lives of older women of color living and aging with HIV.

Our findings reveal that within our sample, Hispanic and Latino women face numerous challenges in addition to treatment. Though more than a third of them completed high school, and over 50% qualified for government assistance such as Supplemental Security Income, 50% continued to work. In fact, they reported the complex impact of the interactive processes of structural forces on their journeys to access resources.

Among the forces described were structural violence, shame, anticipatory loss, and impression management. The complicated intersections of these factors in the multiple levels of the individual’s environment, i.e., individual, community, social, and structural, and across time, have received little attention in the process of healthcare utilization. They provide in-depth contextual material that alters the individual’s ability to access services or the manner in which they utilize available services.

Contextual material is particularly relevant given the many intersecting standards set for women in our society. The intersecting vectored standards, e.g., sexuality and maternity, also include economic factors such as race and poverty. For example, throughout history, black women in the United States have been marked as the source of generational poverty [44,45]. Nevertheless, another vector comprises tropes concerning women of color’s sexuality. Whether seen as insatiable or lascivious [46], women of color have historically been expected to bear these representations in silence. Whether designed to police the bodies and lives of women of color, manipulate public sentiment concerning government spending, or other regulatory goals, these standards, women’s feelings, or the violation or responsibility for violating them may result in profound feelings of shame. Goffman [47] noted that society marks some individuals as “tainted” (p. 3), and for quite some time now, stigma has received substantial focus as a factor impacting the testing and effective treatment of people living with HIV [48,49]. Such demarcations give rise to discriminatory treatment, neglect, and prejudice. Much of this research has focused on reducing the stigma associated with HIV, such as building resources to cope with enacted and perceived discrimination [49]. Shame remains an element of HIV prevention and treatment that remains underexplored—often conflated with internalized self-stigma—defined as the acceptance of negative attitudes from others [50].

However, Bennett and colleagues [51] argued that shame, which has been subsumed into the discussions of stigma, should be given more considerable attention. In their review, Bennett and colleagues proposed that shame, as emergent from stigma, may be a better predictor of depressive symptoms. Early research posited that shame is rooted in the individual’s belief that there is some deficit of the core self [52,53]. Conflating shame with self-stigma neglects the individual’s belief that they are responsible for some violation of the standards set by one society [54]. Furthermore, such conflation strips contextual material when examining the lives of marginalized communities. In this study, participants discussed the emergence of shame in the wake of systemic patterns of structural violence.

Subject to these exposures to violence, participants in this study describe enacting methods to mitigate the shame or to reduce the sense of loss. For this study, we use the term “anticipatory loss”. Participants described retreating or avoiding disclosing. Goffman [55] suggests that after a performance, an actor has the ability—and need—to retreat backstage to a collegial team or to affinal ties where masks are not necessary because the same information and shared understandings already exist. This home group also helps the individual to reflect upon, reinterpret, and refine a necessary front or mask while providing the nurturance and affirmation necessary for the actor to continue to perform. Hence, at the group level, both the home group (i.e., other homeless persons) and audience (i.e., hospital staff) protect the performer’s “self-distantiation”, or the process by which a person comes to feel estranged from himself” because pertinent performance information is repressed or obscured. The home group serves not only as a reference group but as an effective barometer of what an individual is ready to know and a means to test new versions of the “front”. Hence, the narrative must surface the role both the home group plays in terms of the provider’s sense of self and how encounters with consumers threaten or protect this sense of self.

John Rolland [56] identified the term anticipatory loss to describe “the experience of living with possible, probable, or inevitable future loss” [56] (p. 140). Rolland’s concept acknowledges the increased access to and awareness of genetic risk factors and their impact on the individual’s wellness as well as on immediate and future generations. Furthermore, Rolland suggested that such recognition challenged traditional theories of loss, most notably that of Kubler-Ross. Recently, Hosken and Greene [57] have examined anticipatory loss in the context of decision-making among young women who live with BRCA1/2 gene mutations, implicated in the elevated lifetime risk of breast and ovarian cancer. More recently, researchers [58,59] have deployed the concept to examine the experience of parents of children with advanced diseases such as cancer. Moreover, others have examined the anticipatory loss concerning outcomes for children facing the terminal phase of their parent’s illness [60] or parental pre-deployment [61], where extended separation and potential injury, or death, may occur. Despite the variation in context, each author contends that anticipatory loss comprised uncertainty and fear. Much attention is given to the anticipation of future loss and not the cumulative impact of loss across the life span, to which the addition of a significant medical illness further depletes already meager coping strategies and risk reduction methods. In both cases, retreating or avoiding opportunities for engagement are missed.

The participants in this study spoke resolutely of the need for responsiveness on the part of organizations that seek to partner in care with women of color. Through a close intersectional exploration with the women in this study as they engage the system in their lives with family, neighbors, employers, institutions, community-based organizations, clinics, housing agencies, and other implicit actors—such as bias, discrimination, and ageism—their voices reveal a complicated set of strategies for endurance. Such endurance is critical to the performance of their sojourn.

Chief among the emergent needs is the requirement that care must be grounded in a thorough assessment of structural violence and its associated trauma. Traditional therapeutic methods that seek to disrupt the adaptive responses, enacted as responsive to trauma, must negotiate their protective facets. For example, customary models view isolation as contributing to poor mental health outcomes. However, in some cases, participants describe isolation or some level of isolation, e.g., spending time only with family and avoiding groups, as protective. In these confined spaces, women can assemble a feeling of security against the sometimes-chaotic nature of the multiple interactive facets of the social environment.

Our theoretical frame recognizes intersectionality as an interactive and dynamic set of interactions between the individual, her community, and the larger social structure, presently and across time, that can outline the complexity of the lives of women living with HIV. This theoretical frame provides a way to describe the multiple kinds of oppression and social hierarchies that affect women in general and women of color living with HIV and other chronic illnesses.

While critics have derided intersectionality as relativistic, our frame employing the tenets of ecosystem-level (i.e., micro-, mezzo-, and macro-level) data set against histories of institutional and structural violence provides a structure for advancing advocacy and empowerment. These efforts are situated within different women’s circumstances—such as race and ethnicity, class, disability, sexuality, the intersection of work and educational histories, access to resources, housing, food, medical and behavioral health treatments, geography and transportation, and the structural forces of gender-based employment discrimination.

Though this framework holds value for addressing the intersectional factors influencing the lives of marginalized communities, it is critical that we acknowledge the methodological limitation inherent to qualitative inquiry, small samples, and self-reported data. For example, the small sample size from a local healthcare center may not adequately represent the broader experience of older people living with HIV/AIDS. Thus, the project recognized that its findings must be interpreted within the context of a small population of black and Latin older people living with HIV. Furthermore, their recruitment from a healthcare facility may contribute to the variation from those without access to care. Thus, future research must include a large sample that may represent various settings, including those not connected to care. Furthermore, while our participants have been living with HIV diagnoses for many years, our finding may have offered further nuances concerning older people living with HIV/AIDS who were recently diagnosed.

## 5. Conclusions

Social justice demands that service and public health providers advance local and national policies that attend to the inextricable patterns of interactions grounded in racial and gender hierarchies established over time. We recommend that the education of service and public health providers must employ a complex and nuanced analysis in developing and implementing services and interventions that seek to assist women. Models of practice driven by deeply contextualized understandings provide vital tools for assessing singular and cumulative risk and vulnerability. Future research must consider the impact of effective evidence-based, culturally informed training for clinicians. The clinician must have skills in assessing trauma and conducting trauma-informed interventions. Intervention must move beyond linear application to methods informed by observing and responding to the nuanced interactions of the multiple intersecting histories—biological, personal, historical, structural, and global—that inform the lives of women living with HIV. Asserting that interactions of structural, historical, social, and individual forces contribute to the shared experiences of social problems may go some distance in quelling the prevailing partisan social climate of the United States at this time. In pursuit of this goal, the lives of the women in this study underscore just such a gestalt—organized and more than a sum of parts.

## Data Availability

The data presented in this study are available on request from the corresponding author.

## Appendix A

**Table A1:** Demographic statistics.

Gender	n	%
Female	12	100.00
Race/Ethnicity		
Non-Hispanic African American or black	10	83.33
Hispanic or Latino	2	16.67
**Age**		
40–49 years old	1	8.33
50–59 years old	6	50.00
60–69 years old	5	41.67
**Housing**		
In your own house or apartment	11	91.67
In someone else’s house or apartment	1	8.33
**Level of Education**		
<=8th grade	1	8.33
Some high school	4	33.33
High school graduate or equivalency course	4	33.33
Some college or technical school, no degree	3	25.00
**Employment (last 6 months)**		
Yes	6	50.00
No	6	50.00
**Sources of income**		
Unknown	1	8.33
Social Security	1	8.33
Supplemental Security Income or SSI payments or Social Security Disability Insurance (SSDI)	7	58.33
Unemployment compensation	1	8.33
Income from Department of Veteran’s Affairs	2	16.67
**Access to Health insurance**		
No medical coverage	2	16.67
Medicaid	1	8.33
Medicare	2	16.67
Tricare	1	8.33
Private health insurance (e.g., HMO, Blue Cross)	3	25.00
Mixed Medicaid–Medicare	3	25.00
**Rosenburg Health Status Scale**		
Within Normal Limits	12	100.00
